# Ambulatory arterial stiffness index, mortality, and adverse cardiovascular outcomes; Systematic review and meta‐analysis

**DOI:** 10.1111/jch.14755

**Published:** 2024-01-17

**Authors:** Christopher J. Boos, Aung Hein, Ahmed Khattab

**Affiliations:** ^1^ Department of Cardiology University Hospitals Dorset Poole Hospital Poole UK; ^2^ Faculty of Health & Social Sciences Bournemouth University Bournemouth UK

**Keywords:** AASI, ambulatory arterial stiffness index, ambulatory systolic‐diastolic pulse regression index (ASDPRI), cardiovascular death, mortality, stroke and MACE

## Abstract

The ambulatory arterial stiffness index (AASI) is a novel measure of both blood pressure (BP) variability and arterial stiffness. This systematic review and meta‐analysis was designed to evaluate the strength of the association between AASI and mortality and major adverse cardiovascular events (MACE). PubMed, Scopus, CINAHL, Google Scholar. and the Cochrane library were searched for relevant studies to July 31, 2023. Two investigators independently extracted data. The Newcastle‐Ottawa Scale (NOS) was used to assess the quality of all included articles. The relationship between baseline AASI and outcomes were examined using relative risk (RR) ratios with 95% confidence intervals (CI) with RevMan web. Thirteen studies were included and representing 28 855 adult patients who were followed up from 2.2 to 15.2 years. A 1‐standard deviation (1‐SD) increase in AASI was associated with a significant increase in all‐cause death (RR 1.12; 95% CI: 0.95‐1.32), stroke (RR 1.25; 95% CI: 1.09‐1.44), and MACE (RR 1.07; 95% CI: 1.01‐1.13; [*I* ^2^ = 32%]). Higher dichotomized AASI (above vs. below researcher defined cut‐offs) was associated with a significant increase in all‐cause mortality (RR 1.19; 95% CI: 1.06‐1.32), cardiovascular death (RR 1.29; 95% CI: 1.14‐1.46), stroke (RR 1.57; 95% CI: 1.33‐1.85), and MACE (RR1.29; 95% CI: 1.16‐1.44). There was a significant risk of bias in more than 50% of studies with no evidence of significant publication bias. Higher AASI is associated with an increased risk of all‐cause and cardiovascular death, stroke, and MACE. Further high‐quality studies are warranted to determine reproducible AASI cut‐offs to enhance its clinical risk precision.

## 1 INTRODUCTION

Hypertension is one of the strongest risk factors for the development of cardiovascular disease.^1^, ^2^ Ambulatory blood pressure (BP) monitoring (ABPM) has revolutionized the diagnosis and management of hypertension and represents the gold standard for its diagnosis and control.^3^ There is now a vast array of available ABPM measures with several of these being shown to enhance the diagnosis and treatment of hypertension as well as being linked to adverse major adverse cardiovascular events (MACE). These include measures of nocturnal dipping, the early morning BP surge, and measures of BP variability.^4^

First described in 2006 and previously known as the ambulatory systolic‐diastolic pulse regression index (ASDPRI), the ambulatory arterial stiffness index (AASI) has emerged as an increasingly important and novel ABPM measure.^5^ It is distinct from the majority of other ABPM measures in that it is both a measure of BP variability and an indirect measure of arterial stiffness. It is calculated as 1‐minus the regression slope of ambulatory diastolic versus systolic BP. In stiffer arterial trees, the systolic‐diastolic BP regression slope tends to be lower (nearer to 0) and the AASI higher (closer to 1).^5^ The AASI has been shown to vary considerably in cases where 24‐h ambulatory blood pressures and pulse pressures remain similar.^5^ AASI correlates with both pulse wave velocity and the arterial augmentation index as well subclinical markers of target organ damage including carotid intimal thickness, left ventricular hypertrophy and worsening renal function.^6^, ^7^

There is increasing evidence to support a relationship between increasing AASI and MACE as well as all‐cause mortality. There have been two previous meta‐analyses examining the relationship between AASI and adverse clinical outcomes.^8^, ^9^ Together, they reported a significant association between AASI all‐cause mortality, stroke, and cardiovascular events. However, these studies conducted more than 10 years ago and neither the outcome of cardiovascular death nor the relationship between AASI as a continuous variable and all‐cause death were examined. Furthermore, since then there have been several more contemporary studies that have expanded ASSI‐prognosis, evidence base including studies whose outcomes have included cardiovascular death.^10^, ^11^

Hence, we conducted a systematic review and meta‐analysis to investigate the relationship between AASI and clinical outcomes, including all‐cause and cardiovascular death, stroke, and MACE. We aimed to see if contemporary pooled evidence supports a relationship between increased AASI and adverse clinical outcomes.

## 2 METHODS

### 2.1 Search strategy

We conducted a systematic review and meta‐analysis according to a pre‐defined protocol and in accordance to the Preferred Reporting Items for Systematic Reviews and Meta‐Analyses (PRISMA) guidelines and this meta‐analysis has been registered on PROSPERO with no subsequent amendments conducted (https://www.crd.york.ac.uk/PROSPERO; registration ID CRD42023423030).^12^ The primary outcome was all‐cause and cardiovascular death. Secondary outcomes were stroke and MACE.

Five main databases (PubMed) (all fields), Scopus (title, abstract and keywords), CINAHL (all fields), Google Scholar (key words), and the Cochrane library (article title, abstract and keywords) were examined. The main search terms were “Ambulatory arterial Stiffness Index” OR ‘‘ambulatory systolic‐diastolic regression index AND ‘‘Cardiovascular’’ OR ‘‘mortality / death ’’ or MACE OR ‘‘stroke / cerebrovascular event’’ were used to search for all publications from 2006 (when AASI was first defined) to July 31, 2023, and limited to English translation and adults. The reference lists of included studies will also be scanned to supplement the searches and ensure the inclusion of important data sources which might have been missed in our search. All citations were imported into Endnote Version 9 to remove duplicates.

### 2.2 Study selection criteria

This meta‐analysis involved articles that have investigated the association between AASI /ASDPRI and MACE, Cardiovascular death stroke and all‐cause mortality. Relevant articles were only selected if they met the following specific inclusion criteria: (1) Full‐length peer‐reviewed studies; (2) human participants aged ≥ 18 years; (3) Observational studies with a cohort design; (4) the AASI level or AASI cut‐off were reported; (5) AASI was only measured using short‐term ABPM over 24−48 h; and (6) sufficient information was provided for the calculation of risk ratios. Key exclusion criteria were (1) non‐English translated manuscripts; (2) studies reporting on ABPM monitoring; and (3) studies where AASI was measured during or within 1 week of hospitalization for an acute illness. When two or more studies used the same group of original population data, only the articles with the largest sample size and/or the outcome of interest were included. For this systematic review, ambulatory arterial stiffness index is defined as AASI using short‐term ABPM monitoring of 24−48 h.

### 2.3 Data extraction and quality evaluation

The extraction of crucial data and the quality assessment of the study were performed independently by two investigators (CJB and AH) to ensure the accuracy and precision of data extraction. Potential disagreements were resolved through deliberation and intervention efforts of a third investigator (AK) where necessary. The results maximally adjusted models for the outcomes of interest were used where available.

### 2.4 Data synthesis

The details and key characteristics of the eligible studies included: (1) first author and date of publication; (2) Study population (number, age, and sex); (3) Key AASI measurement exclusion criteria; (4) Events Follow‐up period Number and type of events; (4) main reported outcomes; (5) covariate adjustments; (6) Main Outcomes; (7) key results.

### 2.5 Quality evaluation and risk of bias assessment

The Newcastle Ottawa Scale (NOS), developed for case‒control and cohort studies used to assess the quality and risk of bias (ROB) of all included articles.^13^ The NOS grading has three parts: (1) selection, (2) comparability, and (3) exposure and encompasses a total of eight items with a maximum of one star per criteria with two stars for comparability. The total score can range from 0 to 9 stars. Studies with a score of 7−9 were graded as high quality, 4−6 as medium quality, and a score of < 4 poor quality.

### 2.6 Effect measures and statistical analysis

The risk estimates for each study were reported as a hazard ratio (HR), relative risk or risk ratios (RR), odds ratio (OR), or as frequency data based around medians, tertiles quartiles and quintiles with dichotomous expression (above or below distinct cut‐off AASI values). Pooled results were reported as absolute AASI difference (1 standard deviation [1‐SD]) or as the RR (using the inverse variance method) in relation to values above versus below as specific AASI cut‐off. Because no uniform cut‐off values are available for AASI, we reported the RR of “high” versus “low” AASI groups where dichotomous AASI data was reported. We reported the pooled RR separately for all‐cause mortality, cardiovascular death, fatal and non‐fatal stroke, and MACE.

Data was analyzed using Review Manager Web (RevMan Web [Version 5.8.0; The Cochrane Collaboration, (available at revman.cochrane.org]). Heterogeneity was evaluated using the Cochran's Q‐test and *I* ^2^ statistic. Heterogeneity was graded as low, medium, and high for *I* ^2^ scores of 0−25%, 25−50%, and > 50% respectively. Pooled effect sizes were calculated using random‐effects where the effect size was significant (*p* <0.10 or *I* ^2^ > 50%), while fixed‐effects model were used in the absence of significant heterogeneity (*p* > .10 or *I* ^2^ < 50%). Funnel plots were used to detect publication bias. Two‐sided *p* values were reported and a *p* < .05 was considered statistically significant for all analyses. Sensitivity analyses were conducted on pooled analyses excluding the studies of dichotomized AASI using upper tertile or quartile cut‐offs.

## 3 RESULTS

### 3.1 Study selection, characteristics, and risk of bias

**FIGURE 1:**
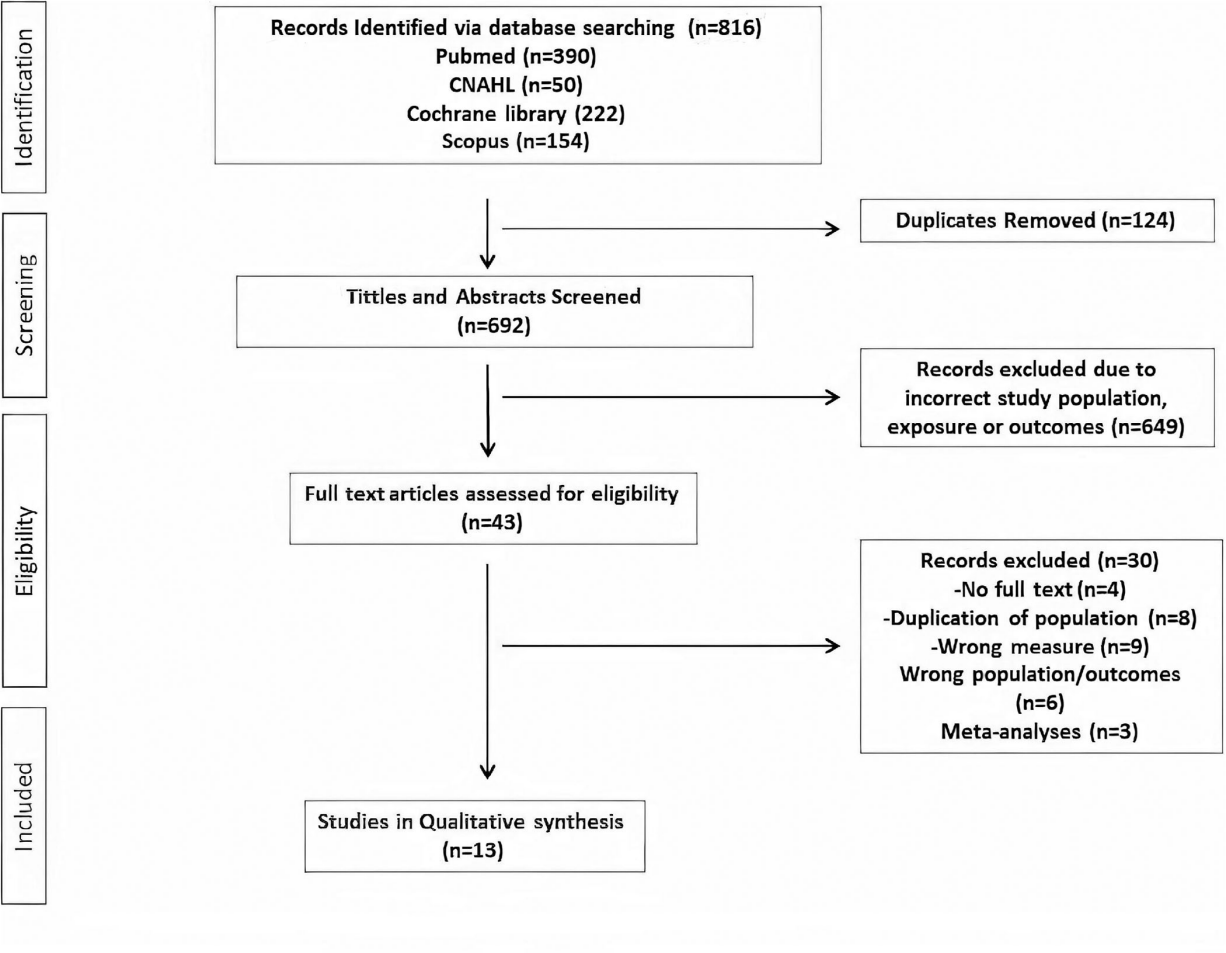
PRISMA Flow chart.

Our initial searches identified 816 potentially suitable publications. Following removal of duplicates and obviously unsuitable studies using the title and abstract search, 43 full text articles were reviewed. From this, a total of 13 studies were included in our meta‐analysis and consisted of a total of 28 566 adult patients (Figure 1). The sample sizes of the included studies ranged from 80 to 11 291 patients. The details of the included studies are shown in Table 1. The average follow‐up ranged from 2.2 to 15.2 years.

**TABLE 1:** Description of included studies.

Author year	Sample size	Population	Exclusion Criteria	Age (y)	Follow up duration (y)	Events	AASI Regression Model	Adjustments	Results
Dolan 2006^24^	11291	General Irish population > 70% untreated HT 53% men	<10 day‐time and < 5 night‐time readings	54.6 ± 14.6 (range 16−96)	5.3 (median)	566 CV deaths including 151 stroke deaths	Continuous and dichotomous (AASI∼0.45)	Age, sex, MAP, BMI, smoking, DM, PP, and history of CVD	↑ adjusted continuous AASI sig linked to stroke death. ↑dichotomised AASI associated with ↑CV (HR 1.59; 95% CI 1.05‐1.27) and stroke deaths (HR 2.42; 95% CI 1.58‐3.72)
Hansen 2006^25^	1829	Danish Community aged 40−70 y 43.5% HT 53.1% men	<14 day time and < 7 night‐time readings	55.5 ± 10.7	9.4 (median)	219 total deaths including 94 CV deaths; 212 MACE (CV death, CHD and stroke, severe PVD)	Continuous and mean (0.56)	Age, sex, MAP, BMI, smoking, TC/HDL, DM and history of CVD	1 SD AASI (0.14) in AASI (*HR 1.62; 95% CI 1.14‐2.28) and AASI > 0.56 (2.51; 95% CI 1.26‐5.0) associated with significant increased risk of stroke
Gosse^26^ 2007	469	Hypertensive French population 40% women Treatment naïve HT	<80 valid measurements	54 ± 14	5.8 ± 3.3	62 MACE (ACS, stroke, sudden death renal or HF, ; 13 CV and 9 non CV deaths	Tertiles T3 0.71 T2 0.54 T1 0.39	Age, sex and 24‐PP for MACE	Age (not age and PP) ‐adjusted AASI (T3 vs. T1) associated with ↑CV events (RR 2.8; 95% CI 1.3‐5.8). No relationship with all‐cause mortality
Kikuya 2007^27^	1542	Japanese General population > 40 years 36.6% men; 62.2% HT	<8 hours awake recordings and < 4 hours during sleep	61.7 ± 10.7(range 40−93)	13.3 (median)	126 CV, 63 stroke and 345 all‐cause deaths	Quartiles (Q) Q4 (> 0.51) vs. Q1‐3	Age, sex, MAP, BMI, smoking, alcohol, DM, and history of CVD	NS Trend to ↑ all‐cause (*HR 1.16; 0.98‐1.38) and CV (*HR 1.29; 95% CI 0.97‐1.70) and stroke (*HR 1.40; 95% 0.94‐2.08) deaths in AASI Q4 vs. Q1‐3
Ben‐Dov 2008^28^	2918	Israeli general population 45% men 59% HT	<15 valid measurements	56 ± 16	7.0 ± 3.6	215 all‐cause deaths	Continuous, median (0.48)	Age, sex, DM and HT, 24 h SBP, and PP and SBP dipping	↑AASI non‐sig associated with all‐cause death (*HR 1.14; 95% 0.95‐1.37)
Palmas 2009^29^	1178	US Medicare diabetics ≥ 55 years 41% men	< 6 daytime and < 4 night‐time readings	∼71	6.6 ± 0.4	287 deaths	Tertiles (T) but AASI cut‐offs not defined	Age, sex, smoking, duration of DM, HF, MI, HDL, ACE/ARB use, office BP and HR	T3 vs. T1 AASI associated with ↑all‐cause death (*HR 1.36; 95%1.01‐1.83)
Muxfeldt 2009^30^	547	Brazilian resistant HTs 71% women	<10 nocturnal readings	65.9 ± 11.23	4.8 (median)	101 MACE (acute MI, sudden death, severe HF, stroke, severe renal failure or PVD); 65 all‐cause deaths including 45 CV deaths	Dichotomous (median 0.55) and continuous	Age, sex, BMI, smoking, DM, HDL, creatinine, number of anti‐hypertensives, history of CVD, and systolic and diastolic BP	1‐SD † in associated increased CV events (⁎HR1.46; 95% CI 1.12‐1.92) but not all‐cause and CV deaths. Unadjusted AASI ≥ 0.55 associated with significantly † MACE and borderline † in CV deaths
Bastos 2010^17^	1200	Portuguese HTs with history of CV events 46.2 men	≤85% valid data	50.70 ± 12.7	15.2 (median)	62 –all‐cause deaths and 152 MACE	Continuous	Age, sex, BMI, anti‐HT medication, 24 h PP	No significant association between AASI and ↑ MACE (*HR 1.67; 95% CI 0.46‐5.99) and stroke (*HR 1.10; 95% CI 0.77‐1.58).
Laugesen 2012^31^	108	Danish type II diabetics 50% women	>3 h missing data	56‐60	9.5 (median)	45 broad MACE (10 fatal)	AASI regression results not reported	Age, duration of DM, history of CVD, serum creatinine, smoking, 24‐h MAP. PP and systolic day –night BP ratio and 24‐PP	AASI > median associated with ↑ MACE on Kaplan‐Meier but not on fully adjusted Cox‐regression (results not reported)
Sobiczewski 2013^11^	891	Polish adults ≥ 70% coronary artery stenosis; 69%men	Atrial fibrillation (no recording minimum criteria reported)	63.7 ± 9.4	6.7 (median)	135 ACS events; 71 CV deaths and 259 MACE (stroke, ACS or CV death)	Tertiles (0.15‐0.28, 0.29‐0.34, and 0.34‐0.64	Age, sex, DM, and history of MI and anti‐HTs	↑AASI associated with ACS (*OR 3.6; 1.2‐11.0) and MACE and MACE (*OR 2.5; 95% CI 1.0‐1.2) and all cause death (*OR2.7; 95% CI 1.2‐9.9)
Boos 2021^10^	508	UK Adults investigated for hypertension 48.8% men	<10 day‐time and < 5 night‐time readings; Atrial fibrillation; Aged 18−80	58.8 ± 14.0	2.2 years	39 MACE (CV death, stroke/TIA, ACS or acute limb ischemia) with 7 CV deaths, 14 strokes/TIAs)	Continuous and dichotomous (≥ 0.45) (Median)	Age sex, ethnicity and PP for MACE; Unadjusted for CV death and stroke	No significant association between ↑ adjusted 1‐SD (HR 3.56: 95% CI 0.41‐30.95) or AASI ≥ 0.45 (HR1.07; 95% CI 0.65‐1.77) and MACE.
Viazzi 2019^32^	80	Hemodialysis patients 72.5% men	No minimum reading number reported	67.4 ± 14.1	4.5 ± 1.7	All‐cause death in 40% of population	Dichotomous (median 0.54)	Age, sex, ambulatory systolic BP and dialysis duration	↑ all‐cause mortality in HD patients with AASI > median (*HR 2.62; 95% CI 1.25‐5.52)
Hoshide 2023^33^	6294	47% men Adults with ≥ 1 CV risk factor in general practice	<6 daytime or 3nighttime readings	68.6 ± 11.7	4.8 (Median)	217 MACE (stroke/TIA, ACS and sudden death) including 119 strokes)	Dichotomous (median 0.58)	Age, sex, BMI, smoking, DM, lipids, use of anti‐HTs, office SBP, and history of CVD	AASI ≥ median associated with increased (*HR 1.89; 95% CI 1.13‐3.15) for stroke; NS for MACE (*HR 1.45; 0.99‐2.13)

Abbreviations: AASI, ambulatory arterial stiffness index; ACS, acute coronary syndrome; BP, blood pressure; CHD, coronary heart disease; CVD, CV disease; DM, diabetes mellitus; HR, hazard ratio; HD, hemodialysis, HF, heart failure; HT, hypertensives/hypertension; MACE, major adverse cardiovascular (CV) event; MAP, mean arterial pressure; OR, odds ratio *adjusted; PVD, peripheral vascular disease; Q, quartiles; SBP, systolic blood pressure; T, tertiles; TC/HDL, total cholesterol to HDL cholesterol.

In total, eight studies reported on the relationship between AASI and all‐cause mortality, seven on CV death, eight on MACE, and seven on stroke outcomes. However, the data used to report the outcome of cardiovascular death was performed using the source data for the study by Boos and associates from 2021.^10^ The relative differences in AASI among patients with compared to those without clinical events were only reported for two studies for all‐cause mortality and two for MACE.

The risk of bias ranged from 3 to 8 with four studies having a high ROB, four moderate and five considered at low ROB. The ROB for each included study is shown in Table 2.

**TABLE 2:** Risk of bias assessment.

	Selection				Comparability	Outcome			
Author year	Representativeness of the exposed cohort	Selection of the non‐exposed cohort	Ascertainment of exposure	Demonstration that Outcome of interest was absent at study start	Comparability of the cohorts in design and or analysis	Assessment of outcome	Long enough follow up for outcomes to occur	Adequacy of follow up	Total score
Dolan 2006	⁎	–	⁎	⁎	⁎⁎	⁎	⁎	⁎	8
Hansen 2006	⁎	–	⁎	–	⁎⁎	⁎	⁎	⁎	7
Gosse 2007	⁎	–	–	–	⁎	–	⁎	–	3
Kikuya 2007	⁎	–	⁎	⁎	⁎⁎	⁎	⁎	⁎	8
Ben‐Dov 2008	⁎	–	⁎	⁎	⁎	⁎	⁎	⁎	7
Palmas 2009	⁎	–	⁎	⁎	⁎⁎	⁎	⁎	⁎	8
Muxfeldt 2009	⁎	–	⁎	⁎	⁎	–	⁎	⁎	6
Bastos 2010	⁎	–	⁎	⁎	⁎	⁎	⁎	–	6
Laugesen 2012	⁎	–	⁎	–	–		⁎	–	3
Sobiczewski 2013	–	–	–	⁎	–	⁎	⁎	–	3
Boos 2021	⁎	–	⁎	⁎	⁎	⁎	–	–	5
Viazzi 2019	–	–	–	⁎	⁎	–	⁎	–	3
Hoshide 2023	⁎	–	⁎	⁎	⁎⁎	–	⁎	–	6

The Newcastle Ottawa scoes a maximum of one star per criteria with two stars for comparability. The total score can range from 0 to 9 stars. Studies with a score of 7–9 were graded as high quality, 4–6 as medium quality, and a score of < 4 poor quality.

### 3.2 All‐cause mortality

**FIGURE 2:**
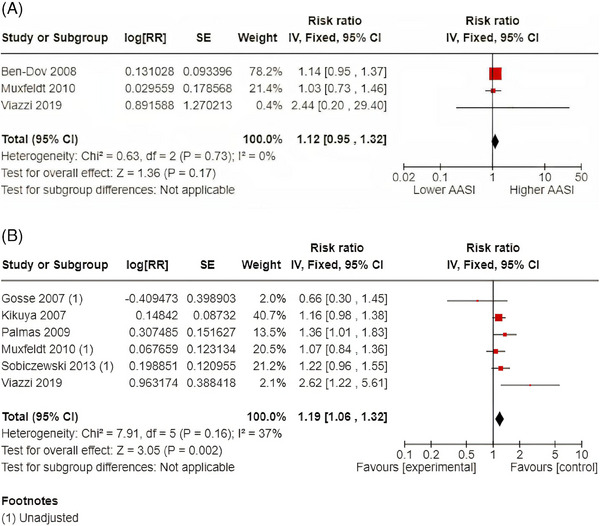
(A) 1‐SD increase in AASI and all‐cause death. (B) Dichotomous AASI and all‐cause death.

There were three studies (3545 patients) that examined the relationship between continuous AASI values and total mortality. A 1‐SD increase in AASI was associated with a 12% increased all‐cause mortality (RR 1.12; 95% CI: 0.95‐1.32 [*I* ^2^ = 0%]). There were six studies (4707 patients) that reported on the relationship between dichotomous AASI values and all‐cause death. The defined cut‐offs were highly variable and included the median, upper AASI tertiles and quartiles. The pooled RR for higher versus lower AASI was 1.19 (95% CI: 1.06‐1.32) corresponding to a 19% RR increase with evidence of moderate heterogeneity (*I* ^2^ = 37%). The pooled analyses for the relationship between AASI and all‐cause death is shown in Figure 2. A sensitivity analysis was performed including only the three studies (1805 patients) that used a median dichotomized AASI and with the removal of the three studies that included upper tertiles or quartiles. Whilst the dichotomized AASI was no longer significantly linked to all‐cause death (RR1.35; 95% 0.95‐1.92), the effect size was maintained but was associated with significant heterogeneity (*I* ^2^ = 64%).

There were two studies (1258 patients) that examined the differences in baseline in AASI and mortality. On a pooled analysis of these two studies (1258 patients), AASI values were significantly higher among the patients who had an all‐cause death versus those who survived (mean difference +0.04; 95% CI: 0.02‐0.06 [*I* ^2^ = 0%]). This pooled analysis is shown in Supplementary Figure 1.

### 3.3 Cardiovascular death

**FIGURE 3:**
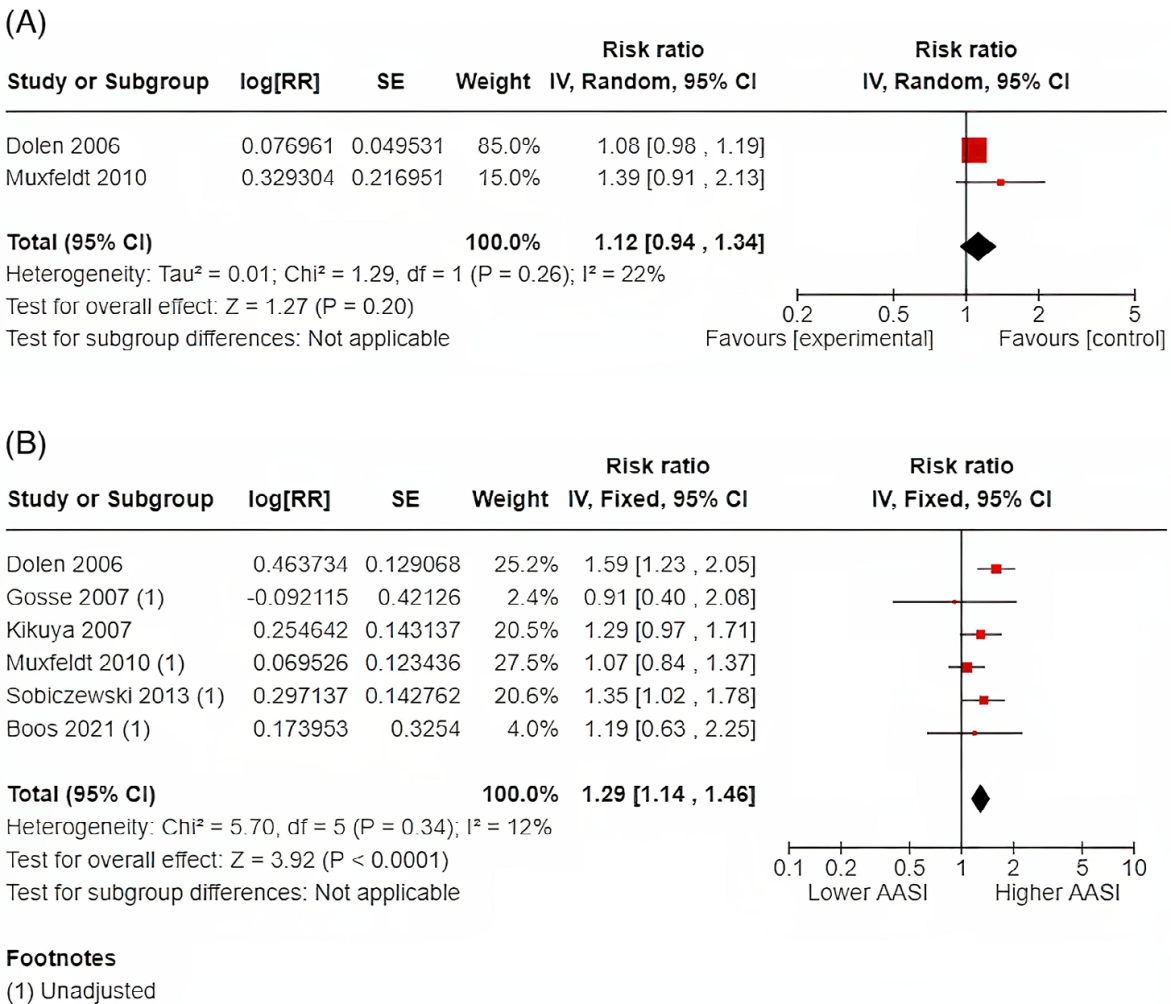
(A) 1‐SD increase in AASI and cardiovascular death. (B) Dichotomous AASI and cardiovascular death.

Only two published studies (n = 1291) to date have reported on the relationship between continuous AASI values and cardiovascular death. The results showed that a 1‐SD increase in AASI was associated with a non‐significantly increased risk of cardiovascular death (RR1.12; 95% CI: 0.94‐1.34 [*I* ^2^ = 22%]). The association between higher versus lower AASI according to defined cut‐off values were available for six studies and included 15 248 patients. The pooled analysis of these studies showed that higher AASI (above researcher determined cut‐offs) was associated with a 29% increased risk of cardiovascular death (RR 1.29; 95% CI: 1.14‐1.46 [*I* ^2^ = 12%]) that was significant. The pooled analyses for the relationship between AASI and cardiovascular death are shown in Figure 3. A sensitivity analysis performed with only the pooled analysis of the three studies (n = 12 346) that used a median (rather than upper tertiles or quartile cut‐offs) was associated with similar effect though the results‐were non‐significant (RR1.29; 95% CI 0.95‐1.73) but heterogeneity significantly increased (*I* ^2^ = 59%). Similar results were found for only the inclusion of fully adjusted models (RR 1.30; 95% CI 10.3‐1.64 [*I* ^2^ = 59%])

### 3.4 Stroke

**FIGURE 4:**
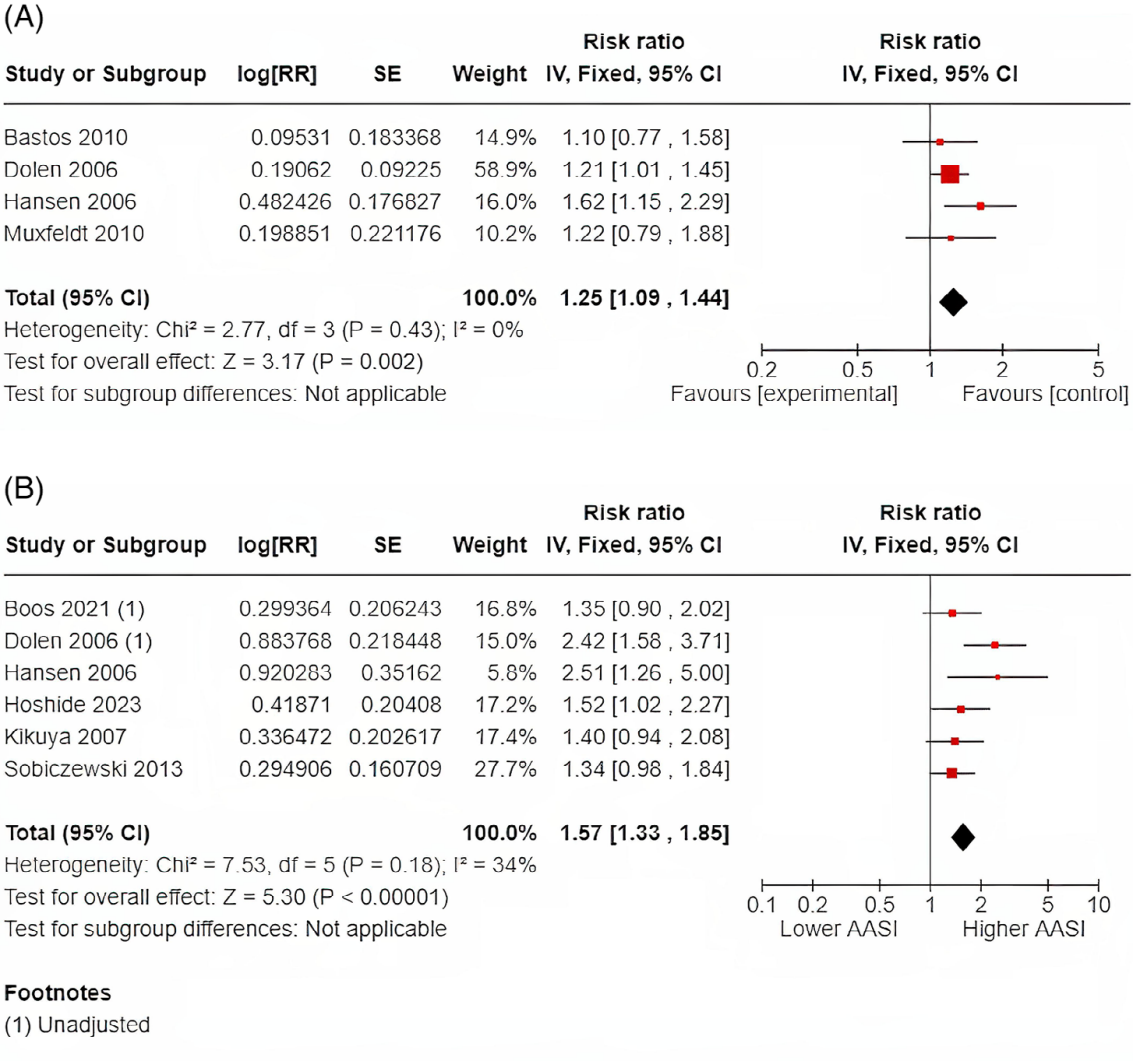
(A) 1‐SD Increase in AASI and stroke. (B) Dichotomous AASI and stroke.

The relationship between AASI, measured as a continuous variable, and stroke was available for four studies consisting of a total of 14 867 patients. Six studies including a total of 10 378 patients investigated the relationship between AASI as a continuous variable and MACE. The pooled analyses for the relationship between AASI and stroke are shown in Figure 4. 1‐SD increase in AASI was associated with a 25% increased risk of stroke (RR 1.25; 95% CI: 1.09‐1.44 [*I* ^2^ = 0%]). The relationship between dichotomized AASI (using medians, upper tertiles, and quartile cut‐offs) was available for six studies and included 22 355 patients. The pooled analysis revealed that showed that higher AASI (above defined cut–off) versus lower was associated with significant and 57% increased risk of stroke (RR 1.57; 95% CI: 1.33‐1.85 with evidence of moderate [*I* ^2^ = 34%]). The results sensitivity analysis including only the four studies (19 922 patients) that used median AASI cut‐offs (not upper tertiles or quartiles) revealed an even stronger relationship between dichotomized AASI and stroke (RR1.75; 95% CI 1.40‐2.20 [*I* ^2^ = 44%]).

### 3.5 Major adverse cardiovascular events

**FIGURE 5:**
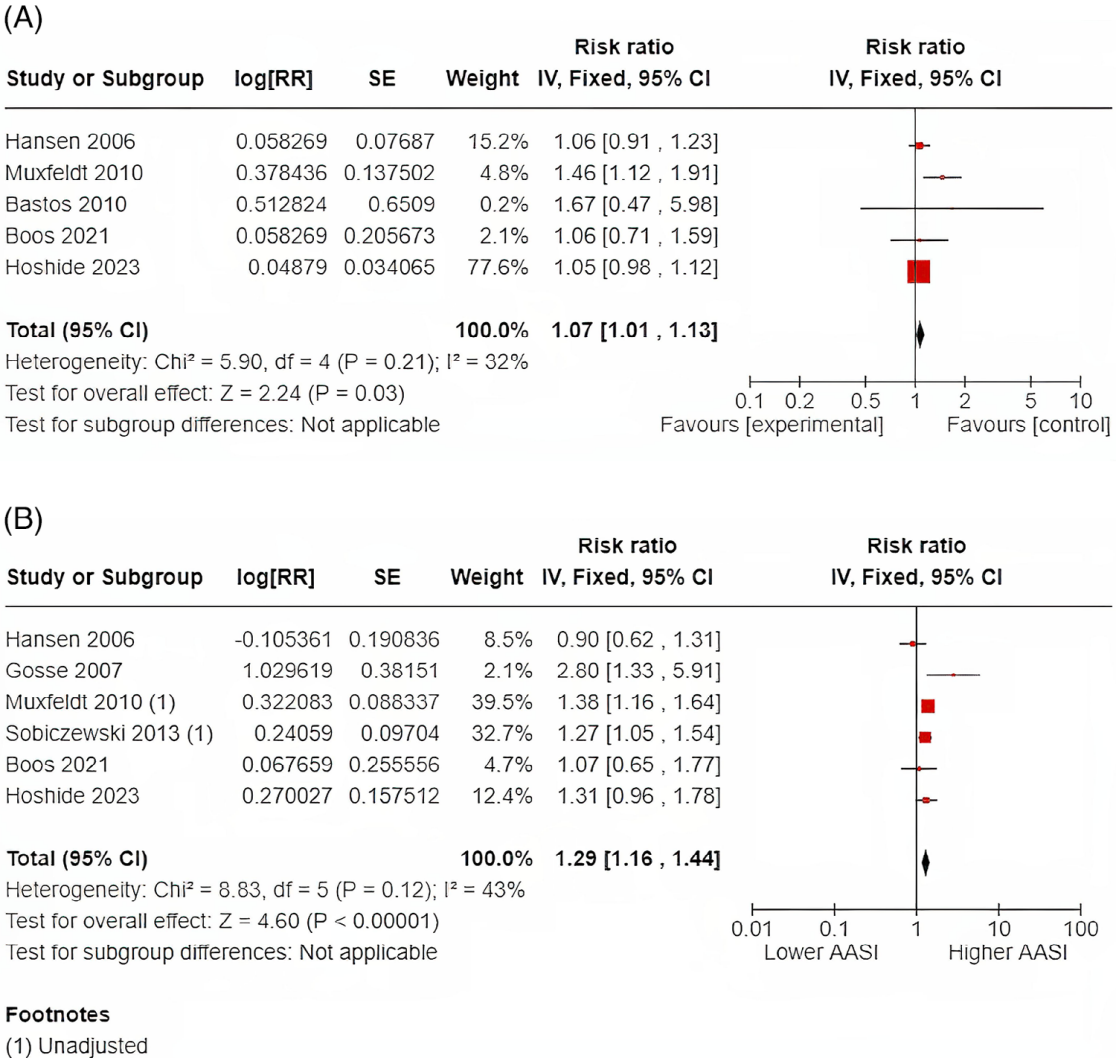
(A) 1‐SD Increase in AASI and MACE. (B) Dichotomous AASI and MACE.

The pooled analyses for the relationship between AASI and MACE are shown in Figure 5. In the five studies that examined AASI, measured as a continuous variable, and MACE, a 1‐SD increase in AASI was associated with a 7% RR increase in MACE (RR 1.07; 95% CI: 1.01‐1.13 with evidence of moderate heterogeneity [*I* ^2^ = 32%)]. In the six studies that examined an AASI as a categorical variable above the defined dichotomous cut‐off, higher AASI was associated with a 29% increase in MACE (RR 1.29; 95% CI: 1.16‐1.44 again moderate heterogeneity [*I* ^2^ = 43%]). In a pooled analysis of the three studies (n = 1085) that investigated mean differences in AASI with future MACE, we found that AASI was significantly higher (+0.08 units; 95% CI: 0.05‐0.10) among the patients that had a MACE versus those that did not (*I* ^2^ = 0%). This pooled analysis is shown in Supplement Figure 2.

### 3.6 Publication bias

Funnel plots examining the risk of publication bias are shown in Supplementary Figures 3–9. The funnel plots showed no evidence of significant asymmetry to support the presence of significant publication bias.

## 4 DISCUSSION

This meta‐analysis consisted of 13 studies and a total of 28 566 patients. We found that a 1‐SD increase in AASI was associated with a non‐significantly increased risk of cardiovascular and all‐cause death and a significant increase in stroke and MACE. Higher versus lower AASI, based on variable researcher defined cut‐offs, associated with a significantly increased risk of all‐cause death, cardiovascular death, stroke, and MACE.

This is the third meta‐analysis to examine the relationship between baseline AASI and clinical endpoints and the first in more than 10 years. The first was published by Aznaouridis and associates and in 2012 and included seven studies.^8^ They found that higher versus lower AASI (using researcher defined cut‐offs) were associated with a significant increased risk of all‐cause death (RR 1.25; 95% CI 1.10‐1.41), stroke (RR 2.01; 95% CI 1.60‐2.52), and MACE (RR 1.51; 95% CI 1.18‐1.93) and a 1‐SD increase in AASI was associated with increased MACE (RR 1.15; 95% CI 1.08‐1.24) and stroke (RR 1.30; 95% CI 1.30‐1.49).^8^ They did not present information on the outcome of cardiovascular death or all‐cause mortality for 1‐SD increase in AASI. In the second meta‐analysis, also published in 2012, Kollias and associates included a total of nine studies.^9^ The authors only examined the outcome of nonfatal and fatal stroke and a combination of MACE and/or cardiovascular death in relation to a 1‐SD in AASI. They observed a hazard ratio for stroke of 1.66 (95% CI 1.48‐1.86) and of 1.09 (95% CI 1.01‐1.18) for cardiovascular death.^9^ In a subsequent publication Kollias and associates also conducted a meta‐analysis, which included a total of only 104 patients, examining the effect of medical treatment on changes in AASI in 2015, but the relationship between AASI to clinical endpoints were not examined.^14^ Their conclusions were that the change in AASI in response to antihypertensive treatment is marginal and remains clinically uncertain.

Our meta‐analysis was the first to examine the relationship between AASI and cardiovascular death and between 1‐SD increase in AASI and all‐cause death. It is also the first study to examine the differences in baseline AASI and all‐cause death and MACE. For MACE the relationship between dichotomous AASI data was significant with a non‐significant trend for a 1‐SD increase in AASI. The results for cardiovascular death were very similar with a significant increased risk with dichotomous AASI and a trend to significance with a 1‐SD increase.

One potentially important source of reporting bias is in the definitions of MACE used in the individual studies. The events that encompassed the MACE definition varied considerably between the studies with the smaller and lower quality studies tending to use broader MACE definition.^15^ With regards to differences in baseline AASI and adverse clinical outcomes AASI were on average 0.04 (mean) and 0.08 units higher in patients who died or had a stroke, respectively, versus the patients who had not suffered these outcomes. This again adds further support to the relationship between increased AASI and all‐cause death and adverse cardiovascular outcomes.

In the two previously cited meta‐analyses^8^, ^14^ the authors reported that there was no evidence of significant risk of publication bias, which is further supported by our latest meta‐analyses. However, the quality of their included studies and the ROB were not graded. In our meta‐analysis of 13 studies, we found that only five studies were of high‐quality and low ROB. Of the remainder four were of moderate ROB and four were graded to be at high ROB. Another important source of potential bias in the data is the use of categorical AASI based along discreet cut‐offs values. There was considerable heterogeneity between the studies not only in terms of the absolute AASI values used to determine specific cut‐offs but also in the categorical distinctions used, which could were variably based on medians, tertiles, and even quartiles. Mean or median AASI categories were used in seven studies with tertiles or quartiles used in four. Compounding this potential source of bias were the marked differences in covariate adjustments between the studies and the trend to less covariate adjustments with dichotomized versus continuous AASI data.

Automated AASI results are included in the summary outputs in an increasing number of ABPM devices,^16^ yet the potential clinical implications of higher values are not widely appreciated, despite the significant relationship between increasing AASI and adverse clinical outcomes. For example, published data have shown a consistently strong relationship between AASI and stroke for which there are several plausible reasons to explain this observation. The AASI is an indirect measure of arterial stiffness and BP variability.^17^, ^18^, ^19^ Increased AASI has been significantly correlated with both arterial augmentation index and pulse wave velocity.^18^, ^20^ Higher AASI is reflective of a weaker correlation between systolic and diastolic BP (hence pulse pressure) suggesting its greater BP variability and is associated with increased arterial stiffness. Increased arterial stiffness and BP variability are strong independent predictors of cardiovascular and all‐cause stroke.^21^, ^22^ Moreover, AASI is known to significantly correlate with age which in itself is a one of the strongest independent predictors of stroke.^10^ AASI values have been shown to be significantly higher in non‐dippers versus normal dippers on 24 h ABPM and even higher in reverse dippers.^10^ AASI has been shown to be an independent predictor of target organ damage including left ventricular hypertrophy chronic kidney disease and increased carotid intimal thickness which are all risk factors for stroke and MACE.^6^ These assets enhance its potential utility as a widely available non‐invasive cardiovascular risk marker.

There was low heterogeneity in six of the measured pooled analyses with moderate heterogeneity in four suggesting important variability in the effect size. The only analyses that generated significant and high heterogeneity population were on sensitivity analyses. This inflation in the measured I^2^ heterogeneity on several of the sensitivity analyses may reflect the reduction in the included study numbers as previously described.^23^  In either case, there is a need for more data from high quality studies particular in relation to the relationship between AASI as a continuous measure and cardiovascular death where only two studies were available.

### 4.1 Limitations

There are several further limitations that need to be acknowledged. Our meta‐analysis included a wide range of adult patient populations including patients with advanced chronic kidney disease, resistant hypertension, and diabetes mellitus. There were three studies with sample size less than 500 patients. Whilst we used the fully adjusted regressions where available for some studies there were no dependent variable adjustment performed, which is another source of potential bias. The included number of studies was relatively small for some of the outcomes and with several studies appearing to be retrospective studies potentially increasing the potential for reporting bias.

## 5 CONCLUSIONS

In this meta‐analysis, which included 13 studies, it was found that higher AASI is associated with an increased risk of all‐cause and cardiovascular death, stroke, and MACE. Further high‐quality studies are warranted and there is a need to develop population dependent reproducible AASI cut‐offs to enhance its use as a cardiovascular risk marker in mainstream clinical practice.

## SUMMARY

**What is known about the topic**

- The ambulatory arterial stiffness index (AASI) is a marker of both arterial stiffness and blood pressure variability.
- The AASI have been linked to target organ damage including chronic kidney disease including left ventricular hypertrophy, worsening kidney dysfunction, and increased carotid intimal thickness.
- The AASI is an increasingly reported automated metric in several commercial ambulatory blood pressure monitor results, yet its utility to influence clinical practice remains uncertain.

**What this study adds**

- Increased AASI is independently associated with an increased risk of all‐cause and cardiovascular mortality, stroke, and major adverse clinical outcomes.
- The average difference in baseline AASI between patients who do and do not develop these adverse outcomes is on average 0.04‐0.08 units higher.
- There is a need to establish clearly defined and reproducible AASI cut‐offs that can be used to enhance cardiovascular risk precision with high accuracy before the use of AASI can be adopted into mainstream clinical practice.

## CONFLICT OF INTEREST STATEMENT

The authors declare no conflicts of interest to declare.

## Supporting information

Supplementary Information

Supplementary Information

Supplementary Information

Supplementary Information

Supplementary Information

Supplementary Information

Supplementary Information

Supplementary Information

Supplementary Information

Supplementary Information

## Data Availability

The data used to conduct this meta‐analysis are all from published manuscripts. The inputs used for the pooled analysis can be made available in response to an appropriate request.
