# Supplementary figures and images for: Ambulatory arterial stiffness index, mortality, and adverse cardiovascular outcomes; Systematic review and meta‐analysis
Source: J Clin Hypertens (Greenwich). 2024 Jan 17;26(2):89–101. doi: 10.1111/jch.14755 (PMC10857461; doi:10.1111/jch.14755)

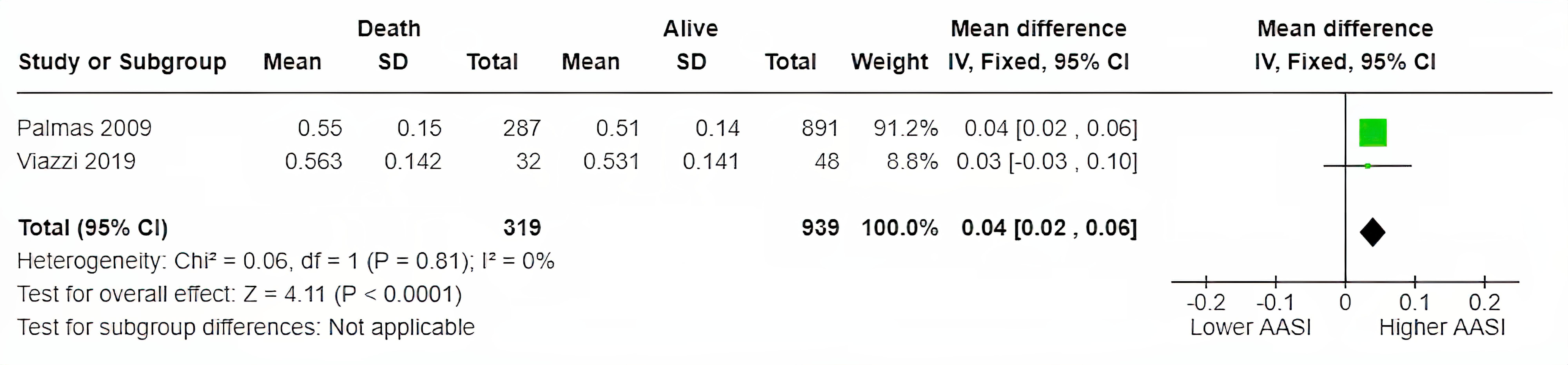

Supplement: Supplementary file 1 — Supplementary Information [file JCH-26-89-s007.jpg]

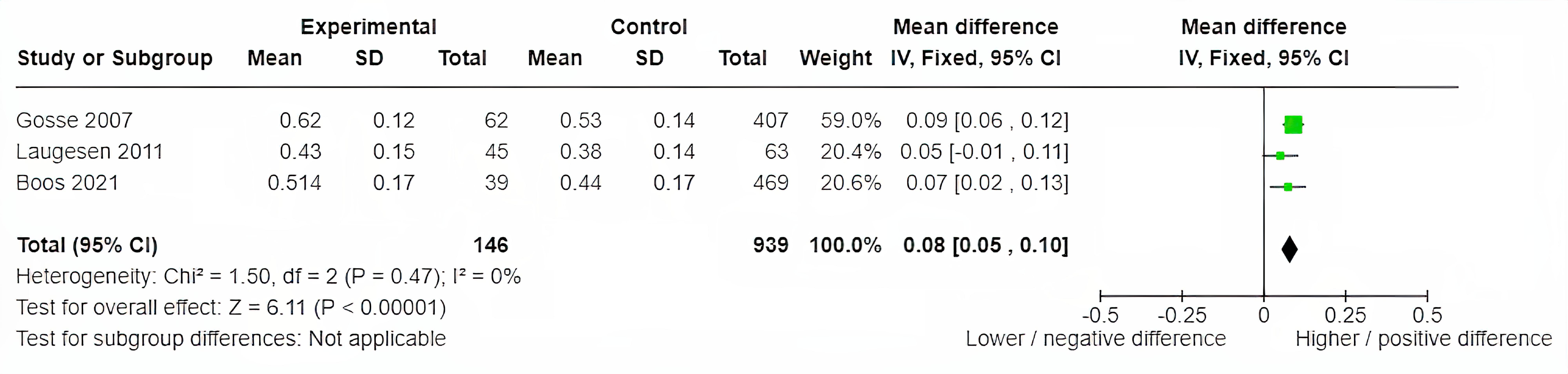

Supplement: Supplementary file 2 — Supplementary Information [file JCH-26-89-s008.jpg]

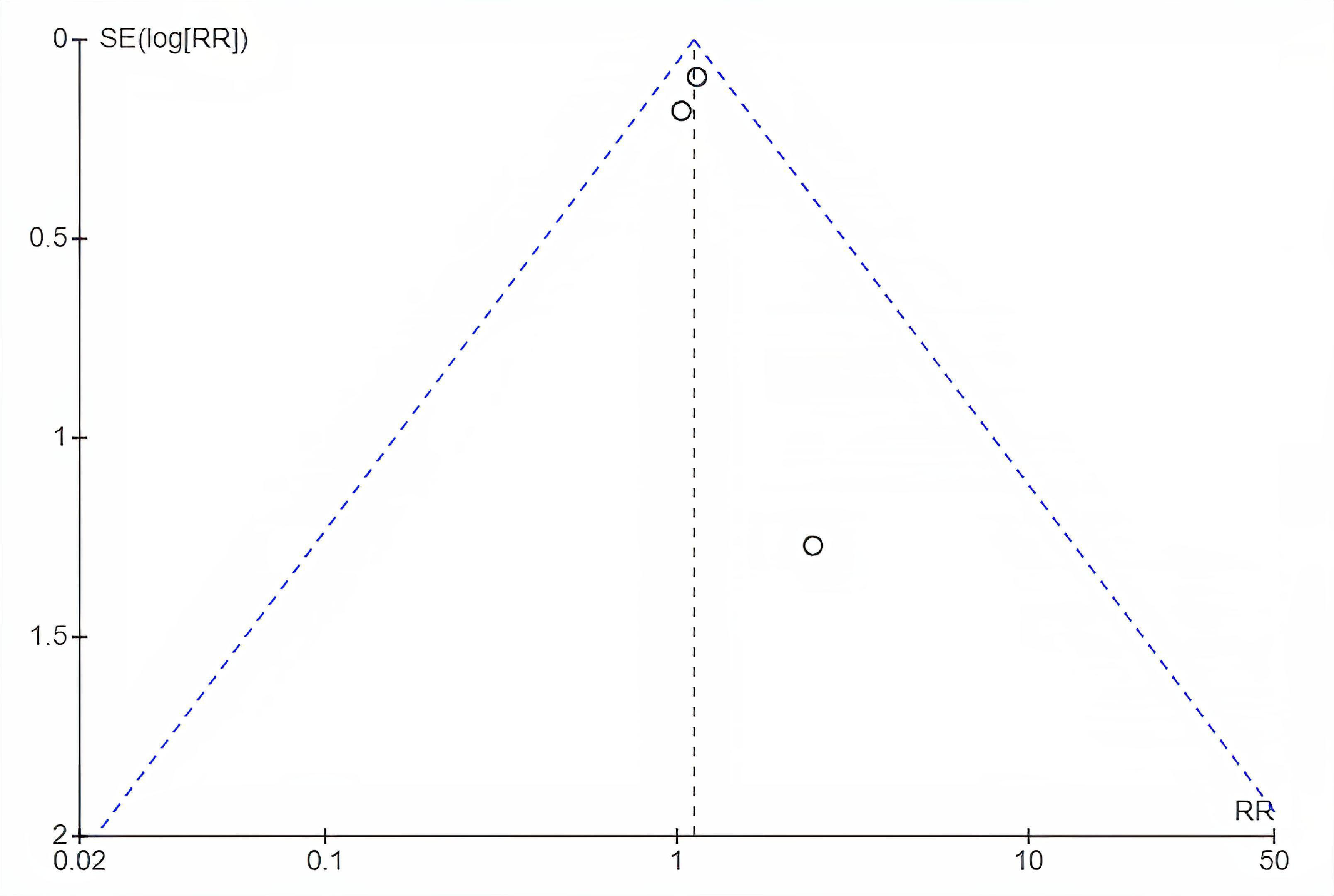

Supplement: Supplementary file 3 — Supplementary Information [file JCH-26-89-s009.jpg]

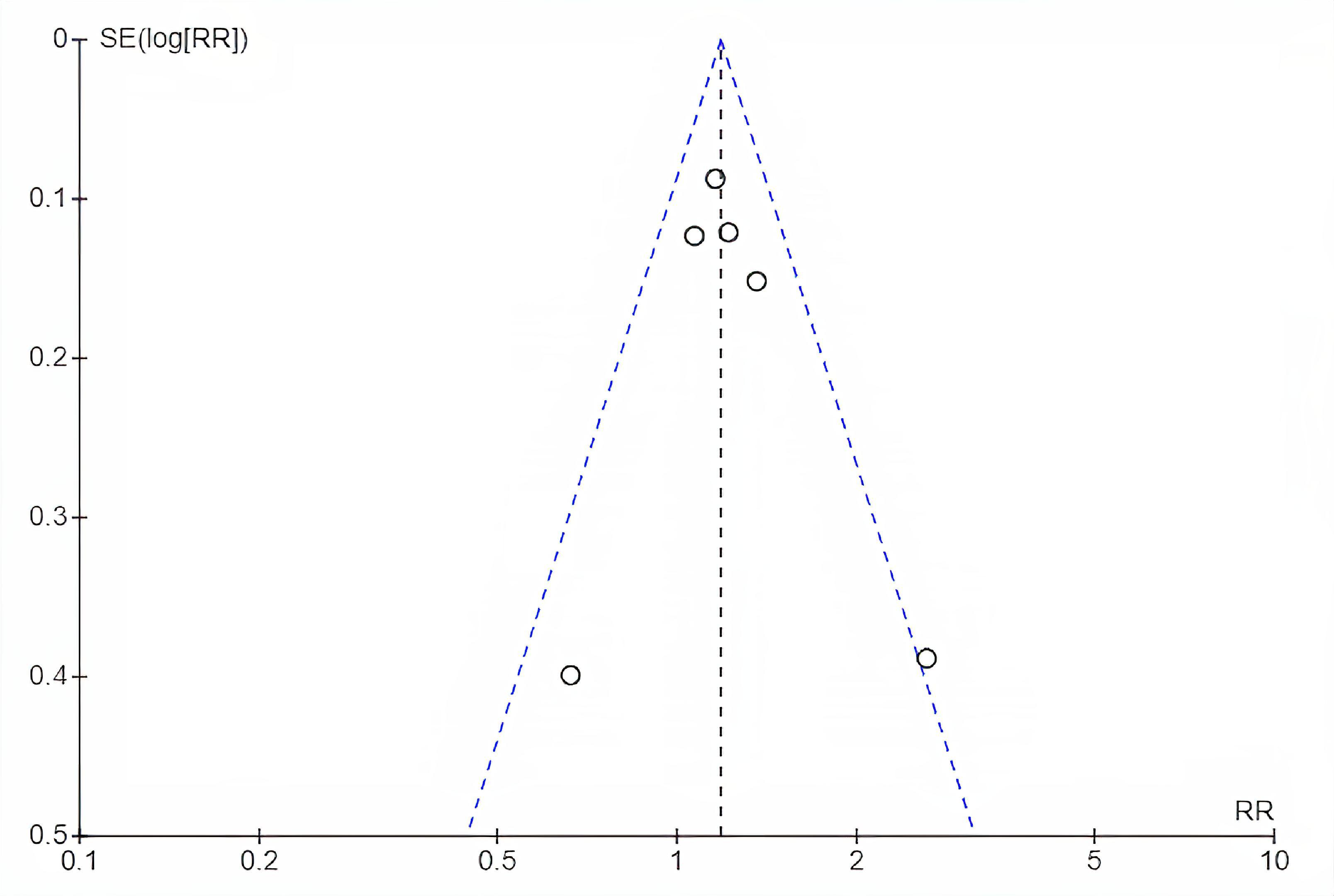

Supplement: Supplementary file 4 — Supplementary Information [file JCH-26-89-s002.jpg]

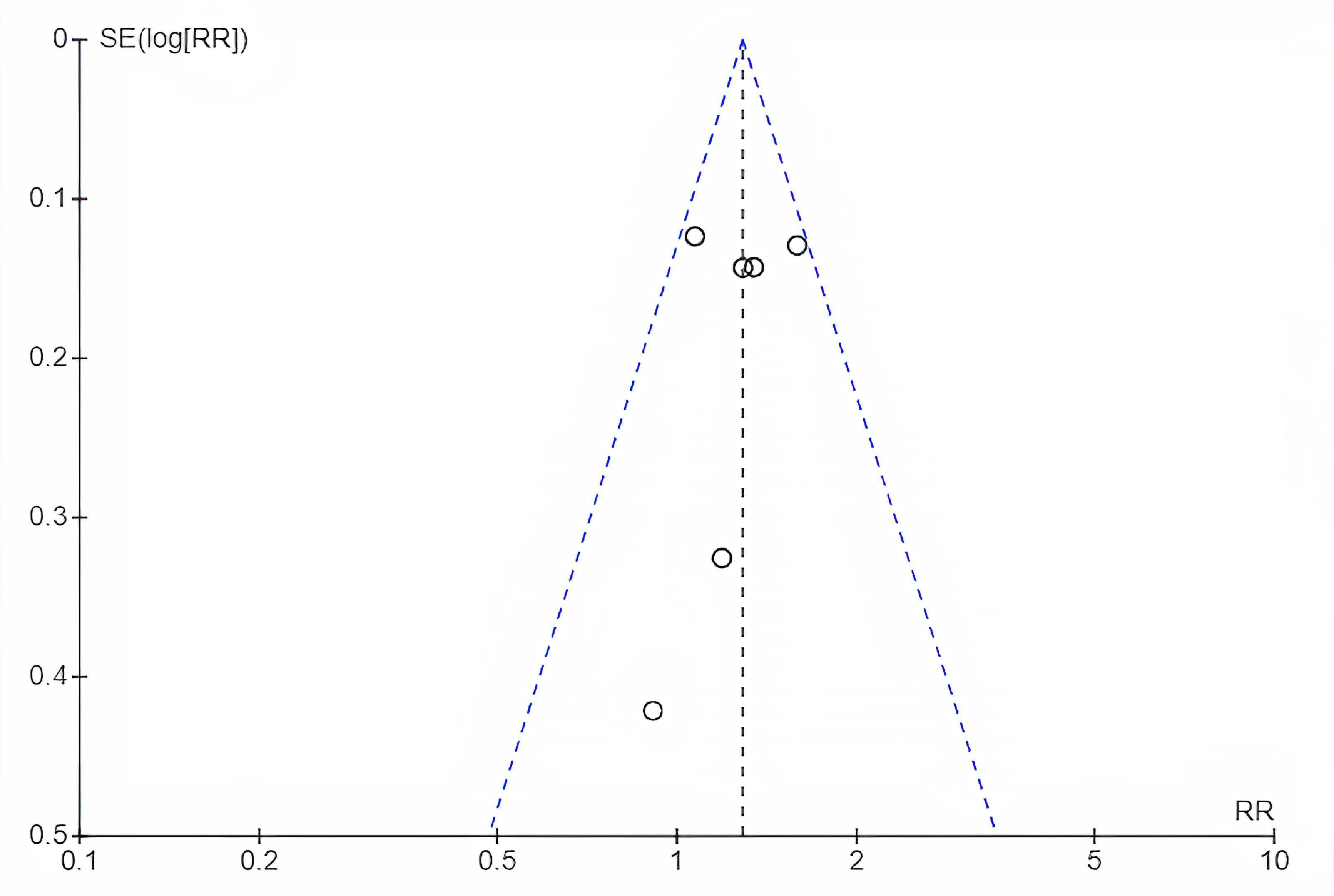

Supplement: Supplementary file 5 — Supplementary Information [file JCH-26-89-s001.jpg]

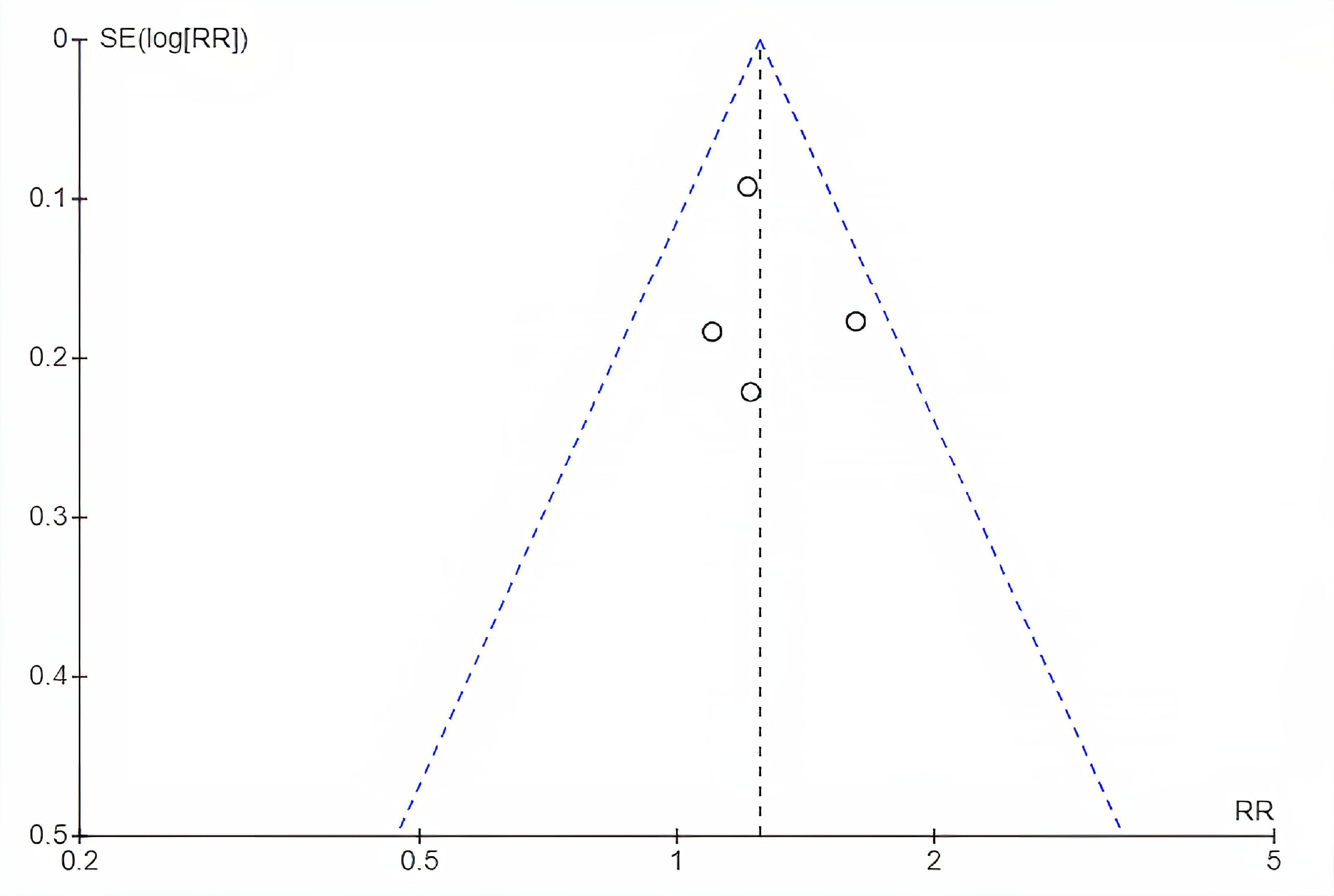

Supplement: Supplementary file 6 — Supplementary Information [file JCH-26-89-s005.jpg]

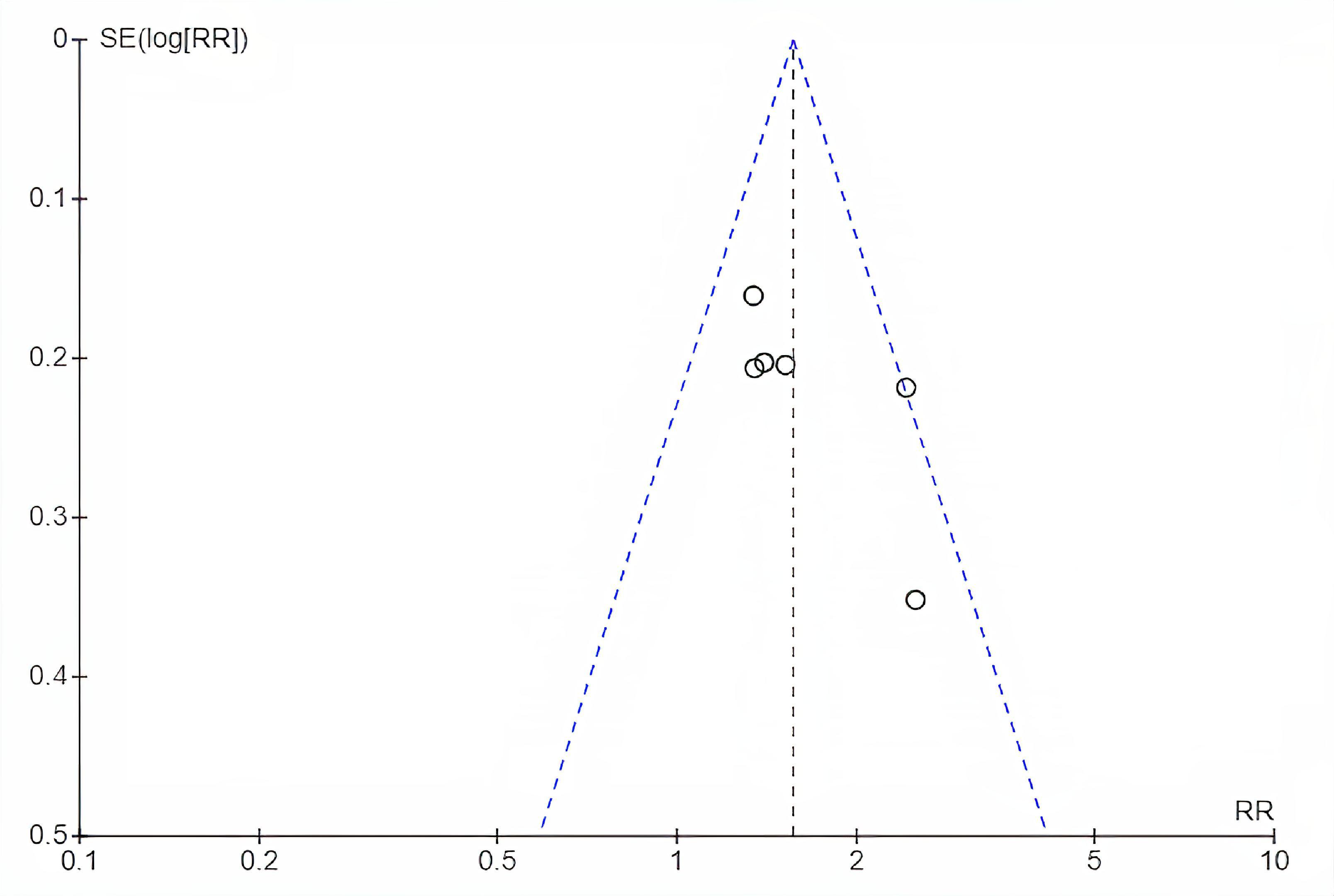

Supplement: Supplementary file 7 — Supplementary Information [file JCH-26-89-s003.jpg]

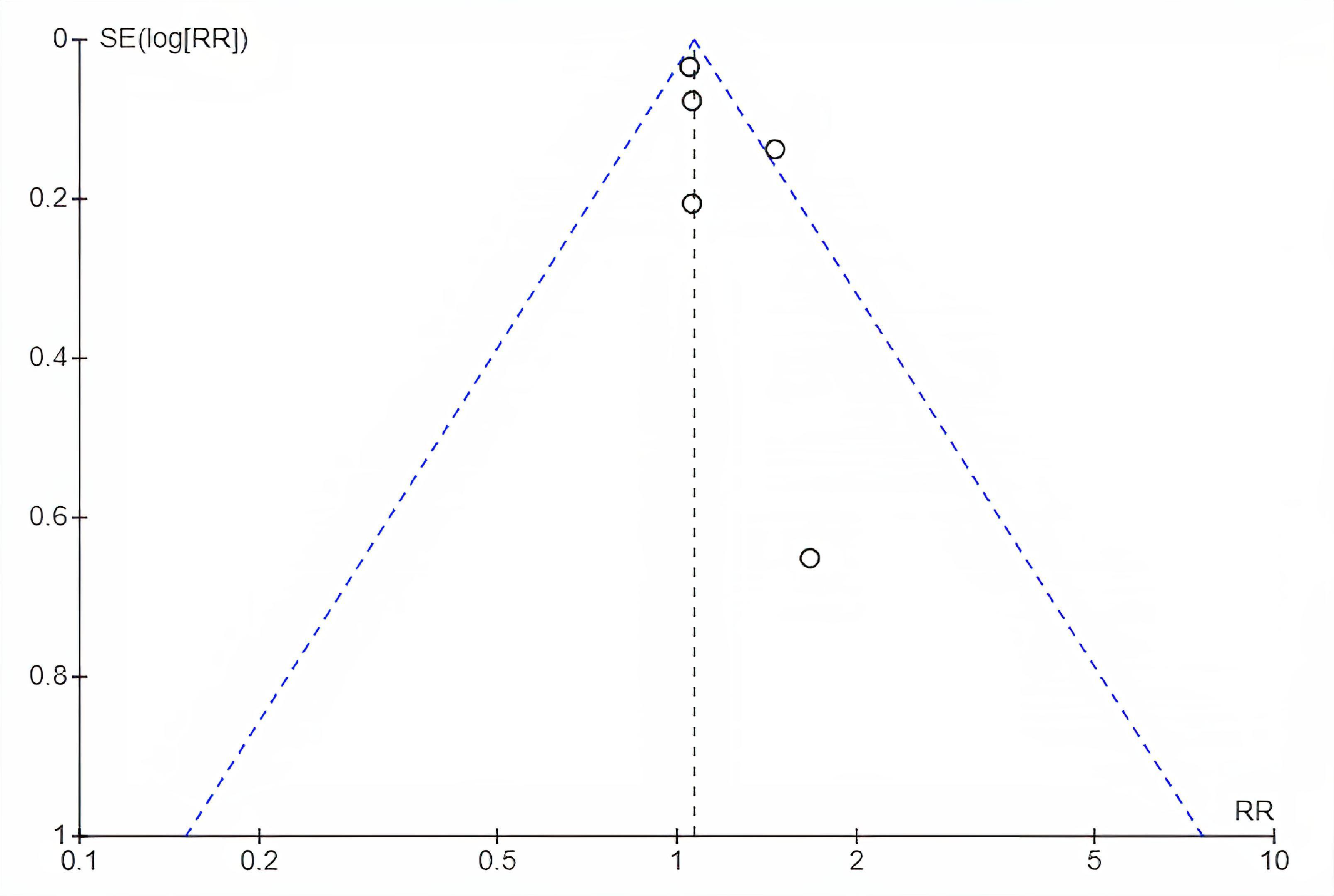

Supplement: Supplementary file 8 — Supplementary Information [file JCH-26-89-s004.jpg]

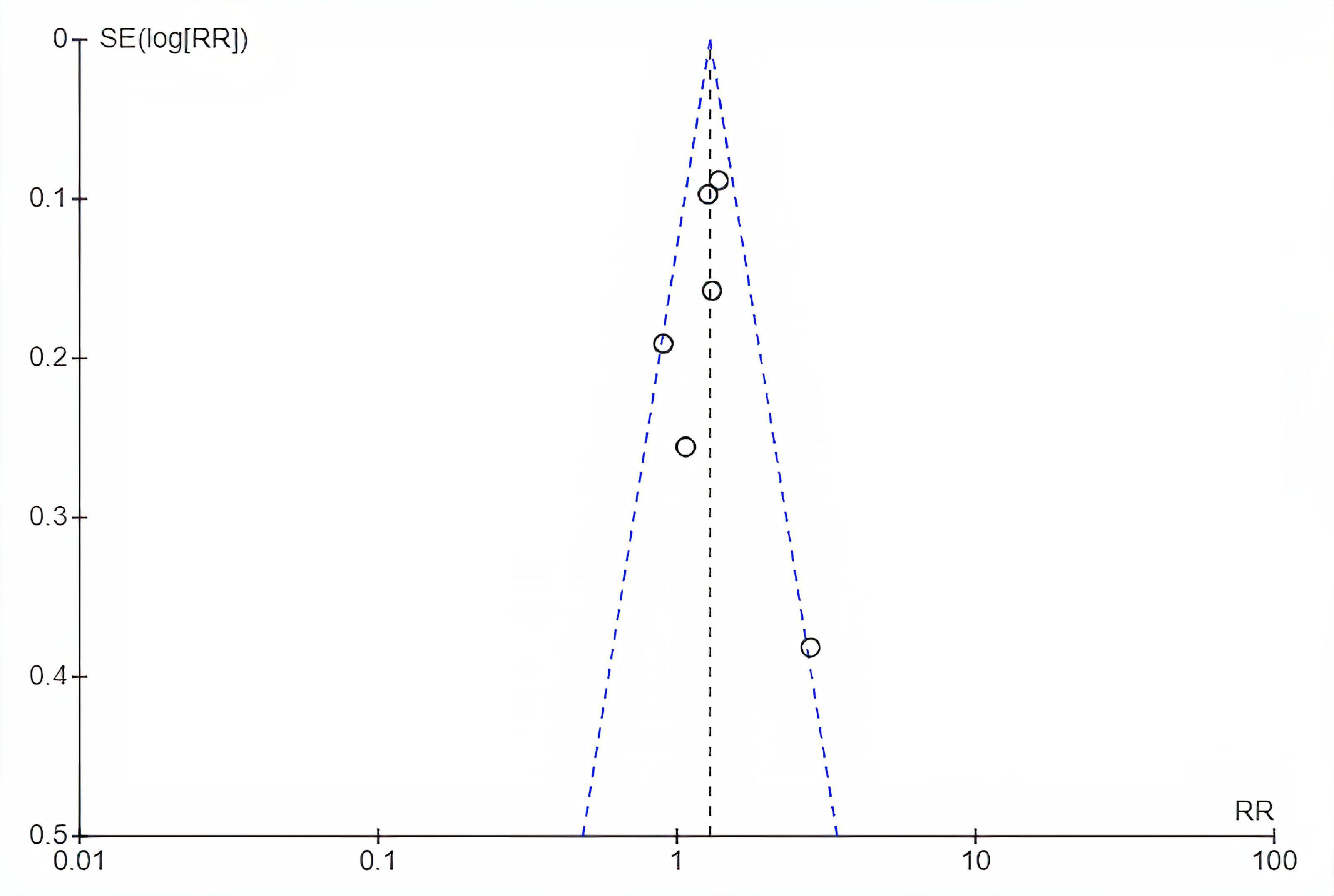

Supplement: Supplementary file 9 — Supplementary Information [file JCH-26-89-s010.jpg]
